# Compound osteoderms preserved in amber reveal the oldest known skink

**DOI:** 10.1038/s41598-024-66451-w

**Published:** 2024-07-08

**Authors:** Juan D. Daza, Edward L. Stanley, Matthew P. Heinicke, Chuck Leah, Daniel S. Doucet, Kelsey L. Fenner, J. Salvador Arias, Ru D. A. Smith, Adolf M. Peretti, Nyi Nyi Aung, Aaron M. Bauer

**Affiliations:** 1 Department of Biological Sciences, Sam Houston State University, Huntsville, TX 77341 USA; 2 Florida Museum of Natural History, University of Florida, Gainesville, FL 32611 USA; 3 University of Michigan–Dearborn, Dearborn, MI 48128 USA; 4 Houston Museum of Natural Sciences, Houston, TX 77030 USA; 5 Department of Biology, University of Florida, Gainesville, FL 32611 USA; 6 Laboratorio de Genética Evolutiva, Instituto de Biología Subtropical, CONICET-Universidad Nacional de Misiones, and Facultad de Ciencias Exactas, Químicas y Naturales, 3300 Posadas, Misiones Argentina; 7 Jabatan Geologi, University Malaya, 50603 Kuala Lumpur, Wilayah Persekutuan Kuala Lumpur Malaysia; 8 Peretti Museum Foundation, Baumschulweg, 13, 6045 Meggen, Switzerland; 9 Myanmar Geosciences Society, Department of Geology, University of Yangon, Yangon, 11041 Myanmar; 10 Department of Biology and Center for Biodiversity and Ecosystem Stewardship, Villanova University, Villanova, PA 19085 USA

**Keywords:** Evolution, Zoology, Anatomy

## Abstract

Scincidae is one of the most species-rich and cosmopolitan clades of squamate reptiles. Abundant disarticulated fossil material has also been attributed to this group, however, no complete pre-Cenozoic crown-scincid specimens have been found. A specimen in Burmite (99 MYA) is the first fossil that can be unambiguously referred to this clade. Our analyses place it as nested within extant skinks, supported by the presence of compound osteoderms formed by articulated small ostedermites. The specimen has a combination of dorsal and ventral compound osteoderms and overlapping cycloid scales that is limited to skinks. We propose that this type of osteoderm evolved as a response to an increased overlap of scales, and to reduced stiffness of the dermal armour. Compound osteoderms could be a key innovation that facilitated diversification in this megadiverse family.

## Introduction

Scincidae is a megadiverse clade of squamates, which today has attained a near cosmopolitan distribution in temperate and tropical regions worldwide^1,2^. It is represented by more than 1745 described living species^3^, comprising nearly 15% of all extant lizards. Typical skinks have cylindrical bodies and relatively short limbs and, in over 50 skink lineages, evolutionary transitions towards limb reduction or loss have occurred^4–8^. Among extant scincoids (Xantusiidae, Gerrhosauridae, Cordylidae, Scincidae), members of Scincidae exhibit the greatest range in body length, including some miniaturized forms having a body length (SVL) of only a few centimetres (e.g. *Scincella macrotis*, 24 mm SVL^9^) to the extinct *Tiliqua frangens*, which may have reached half a meter or more^10^. Skinks also show great variation in the number of presacral vertebrae, ranging from 26 to 108 ^11^ which, in combination with cycloid scales and compound osteoderms, may have facilitated the repeated evolution of fossorial and limbless morphotypes^7,12,13^.

Most skinks are characterized by possession of smooth, cycloid scales underlain by compound osteoderms—bony plates in the dermis that are made up of several articulated “osteodermites” per scale^14^. Molecular and morphological data indicate that skinks are most closely related to girdled lizards (Cordylidae), plated lizards (Gerrhosauridae) and night lizards (Xantusiidae) among extant lizard groups^15–19^. Morphological evidence also supports a close relationship of skinks to several fossil lizard groups, including Carusiidae, Globauridae, and Paramacellodidae^20–22^.

**Figure 1:**
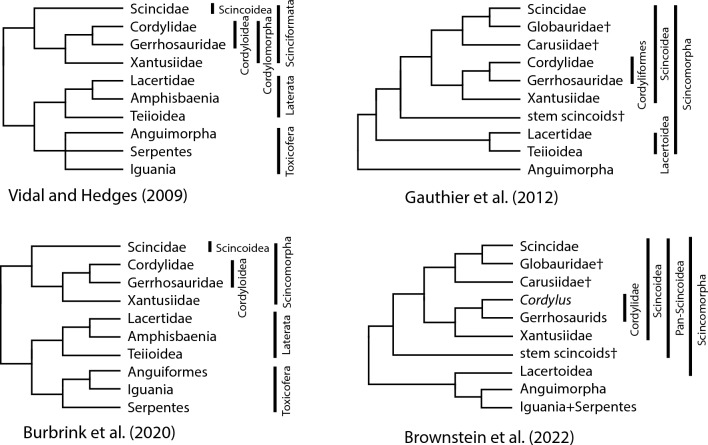
Comparison among higher-level classifications of skinks and relatives as used in recent studies.

There are large discrepancies as to the content and meaning of names applied to higher taxa in classifications of skinks and the relatives listed above (Fig. 1). The variation in usage reflects differences in hypothesized evolutionary relationships, the use of molecular versus morphological characters, inclusion vs. exclusion of fossil taxa in the relevant studies, and the number of ranked and unranked levels applied. Vidal and Hedges^15^ in their molecularly-derived classification of squamates, coined the name Scinciformata to include Cordylidae Gerrhosauridae, Xantusiidae and Scincidae. Hedges^23^ and Hedges and Conn^24^ subdivided skinks (termed by them Scincomorpha) among three superfamilies (Lygosomoidea, Scincoidea, and Acontoidea) and nine families, one of them being the family Scincidae (the sole family within the superfamily Scincoidea). The content and arrangement of these families have been called into question by subsequent, large-scale phylogenetic analyses^16,25,26^. Most other classifications have included all extant skinks in the Scincidae and apply Scincoidea to a group including both skinks and other extant or fossil taxa. The morphological study of Gauthier et al.^20^ used Scincoidea to include Cordyliformes (Cordylidae + Gerrhosauridae), Xantusiidae, Carusiidae, Globauridae and Scincidae, and two “stem Scincoidea” groups (the Middle Jurassic/ Late Cretaceous Paramacellodidae, and the Late Cretaceous *Parmeosuarus scutatus*). A recent analysis^21^ with the inclusion of two tridimensional fossils from the late Jurassic, *Microteras borealis* and *Eoscincus ornatus,* recovered these fossils with all the aforementioned taxa in a largely unresolved total clade Pan-Scincoidea defined as all extinct and extant taxa more closely related to Scincoidea than to other crown squamates, with Scincoidea defined as the crown clade containing skinks, cordylids, and gerrhosaurs. We apply these usages of Pan-Scincoidea, Scincoidea, and Scincidae in this study.

Gauthier et al.^20^ maintained the use of Camp’s Scincomorpha^27^ as defined by Estes et al.^28^, which is a more inclusive group that includes Lacertoidea (Lacertidae, Teiidae, Gymnophthalmidae and Alopoglossidae) and Scincoidea (Cordylidae, Gerrhosauridae, Scincidae, and Xantusiidae plus two fossil families, the Jurassic Globauridae, and Late Cretaceous Carusiidae). In a molecular study of extant squamates, Burbrink et al.^19^ applied Scincomorpha to the clade corresponding to Scinciformata of Vidal and Hedges^15^ or Scincoidea of Gauthier et al.^20^, as lacertoids (Laterata) were found to be more closely related to anguimorphs, iguanians, and snakes.

Much disarticulated material has been referred to Scincidae^29,30^, although many of these fossils are in an uncertain position within the total clade Pan-Scincoidea. The Late Cretaceous and Paleocene *Contogenys sloani*, originally described as part of Scincidae^31^, was subsequently transferred to a new family, the Contogeniidae^32^. No morphological or combined morphological-molecular phylogenetic analysis has included the Mesozoic and Paleogene taxa attributed to Scincidae (*Berruva louisi*, *Penemabuya antecessor*, *Aocnodromeus corrugatus*, *Gekkomimus rugosus*, *Orthoscincus malperiensis*, *Axonoscincus sabatieri* and *Eumeces antiquus*^33–37^) within the crown Scincidae^17,20^. In some cases, phylogenetic assessments have supported the allocation of Neogene fossils within crown Scincidae, as is the case of *Proegernia mikebulli*, a fossil lizard significant for establishing a minimum age for the colonization of Australia by skinks as the oldest known member of Egerniinae^38^.

Broadly sampled squamate time-tree analyses have estimated that crown Scincoidea originated during the middle Jurassic (approximately 170 Million years ago), with the crown age of Scincidae dating to the Cenomanian, in the Late Cretaceous (approximately 94 million years ago)^18,39^. This implies a ~ 75 million-year range spanning the late Cretaceous and Paleogene during which skinks are presumed to have been extant, but no unambiguous Scincidae fossils are known. The paucity of Mesozoic crown scincoid fossils limits our understanding of the origin of skinks, and known Mesozoic scincoid fossils sometimes show mixed characters found in Scincidae (dentary structure) and Gerrhosauridae (tooth morphology), as in the case of *Orthrioscincus mixtus*^40^.

The taxonomy of fossil lizards relies mainly on skull characters. However, the features of dermal armor, commonly preserved in some fossils and sometimes exhibiting outstanding preservation (e.g. Stem Scincoidea^41^, Glyptosaurinae^42,43^, Helodermatidae^44^, Shinisauria^45^, and Anguidae^46^, among others), may also be informative. Within Scincoidea, there is a remarkable pattern of distribution of body armor; while osteoderms are common in Scincidae, Cordylidae and Gerrhosauridae, they are absent among Xantusiidae, including some amber embedded lizards with excellent integumentary preservation^22^.

The distribution and structure of ossified dermal armor among Scincoidea is highly variable^47^, but cycloid, compound osteoderms, encircling the body, occur only in Scincidae^14,27,48^; ventral compound osteoderms are also found gerrhosaurs (two gerrhosaurid species also possess dorsal compound osteoderms—*Gerrhosaurus skoogi* and *Cordylosaurus subtessellatus*). Among fossil taxa, only *Parmeosaurus scutatus* shows some ventral compound osteoderms or duplex osteoderms, formed by two units^41^, but these are more rectangular and arranged in a grid-like pattern, being similar to the dorsal osteoderms of gerrhosaurs and quite unlike the cycloid, imbricate, staggered arrangement of osteoderms of extant skinks.

In this paper we describe a fossil in Burmite that preserves imbricating cycloid scales with compound osteoderms formed by small articulated osteodermites, structurally identical to those in modern skinks. Although this fossil is missing most of the vertebral column and skull, making it difficult to compare with other fossil taxa available from the Mezosoic, integumentary similarities with extant members of Scincidae, allow us to place this fossil as the oldest known representative of Pan-Scincidae. Currently over 100 specimens of squamates are known from Burmite. In this large sample, this new fossil is the only one that preserves this osteodermal morphology which makes it diagnosable to the Scincidae, and differentiates it from all known fossil squamates from the Cretaceous. The specimen is incomplete, but it does retain both postcranial skeletal elements and integumentary structures and, though less than ideal, it provides a basis for comparison with any putative scincid material that might be found in the future. Given both the significance of its osteodermal condition and the scarcity of substantially complete Burmite fossil lizards (and therefore the low likelihood of finding a more osteologically complete fossil that also retains osteoderms), we here opt to describe this taxon despite its incompleteness.

## Results

Systematic Paleontology.

Squamata Oppel, 1811.

Scincoidea Oppel, 1811.

Scincidae Gray, 1825.

**Figure 2:**
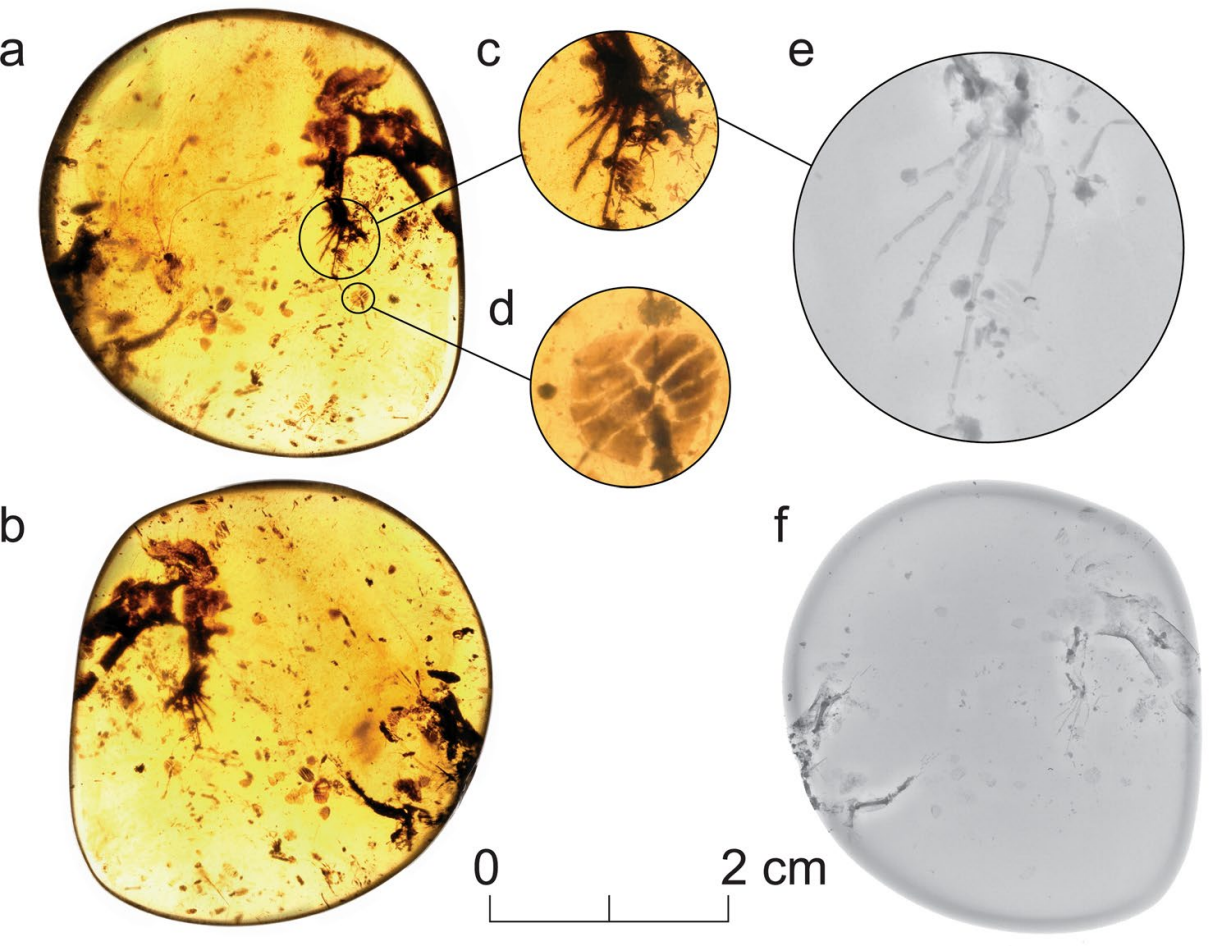
*Electroscincus zedi*. Fossil in ventral (**a**) and dorsal (**b**) views. Detail of the right foot (**c, e**) and osteoderms (**d**). X-ray of the whole specimen showing the skeletal remains, and several articulated and scattered osteoderms (**f**). Scale bar applies to the entire amber piece.

**Figure 3:**
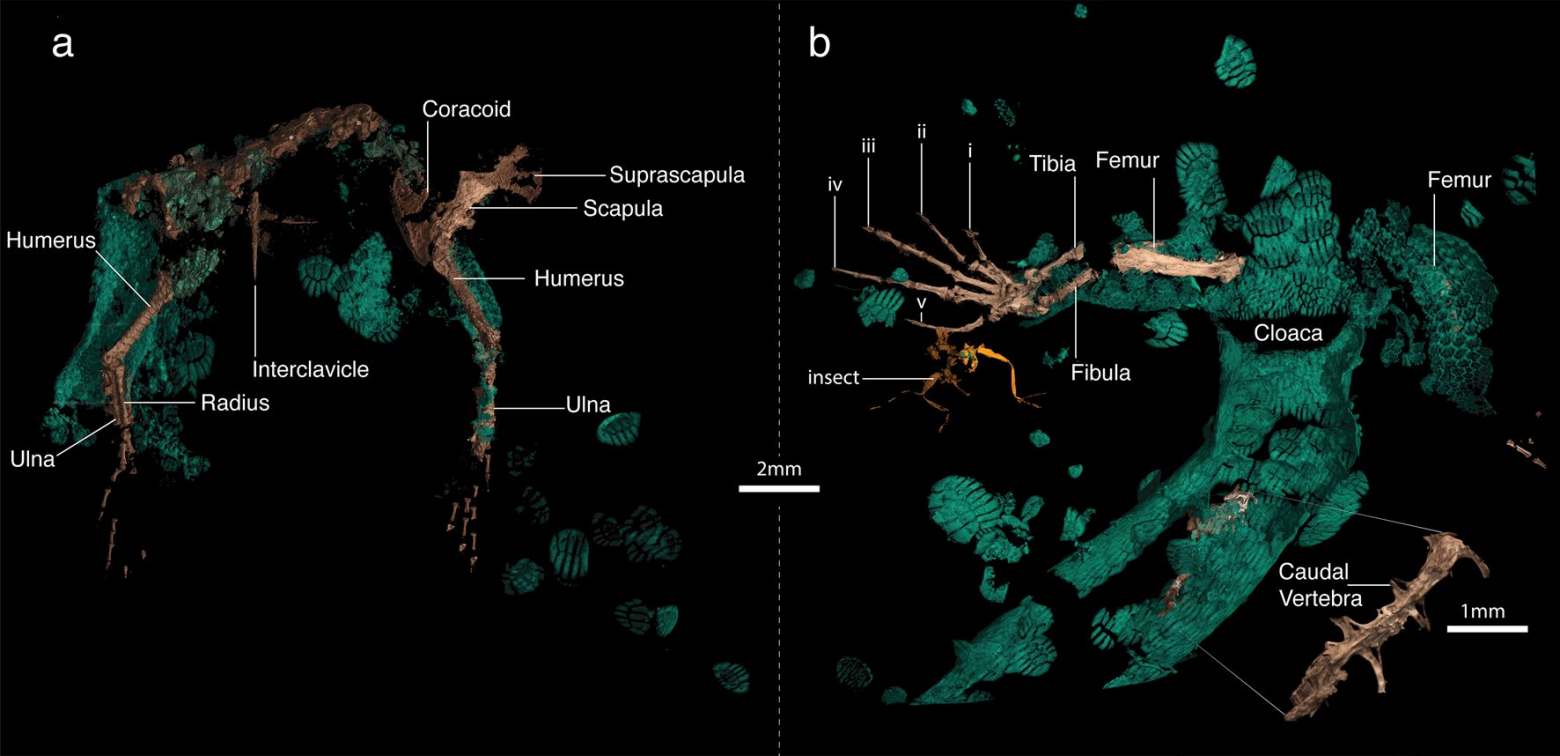
*Electroscincus zedi*, pectoral and pelvic girdle in ventral view (**a, b**). Numbers indicate digit number.

*Electroscincus zedi*, gen. et sp. nov (Figs. 2, 3).

### Diagnosis

A small lizard with an estimated snout vent length (SVL) of 30 mm. *Electroscincus zedi* differs from all other known squamates from the Mesozoic by the presence of imbricate, compound osteoderms arranged in a staggered pattern around the body, supporting its placement in Scincidae (Figs. 2, 3). Its inclusion within Scincidae is also supported by its possession of cycloid scales around the body^28^ overlying compound osteoderms (in some Cordyliformes compound osteoderms are present in scales of the ventral surface only^49,50^). The osteoderms are very different from rectangular and imbricated paramacellodid osteoderms.

When compared with temporally relevant fossil taxa from the Cretaceous that have been associated with the Scincoidea, SVL estimates allow us to distinguish *Electroscincus* from many taxa; we estimated the SVL of *Electroscincus zedi* to be not longer than 30 mm, based on the distance between the pectoral girdle and the cloaca, and assuming the specimen is an adult or a subadult (see description of the skeleton for comments on ossification); estimating SVL in fossil lizards represented by isolated material is difficult, but using the length of bones of a typical living, fully-limbed, non-attenuate skink species (*Emoia pallidiceps* Smithsonian National Museum of Natural History USNM 166276), we can calculate a rough estimate of their size. *Tepexisaurus*, *Myrmecodaptria*, *Carusia*, *Globuara*, and *Eoxanta* are twice or more the SVL of *E. zedi* and *Eoscincus* (North America), *Microteras* (North America) and *Paramacellodus* (England and France) are approximately 17 mm larger than the new species. *Retinosaurus* and *E. zedi* have a similar estimated SVL, and both have a cruciform interclavicle (clavicles not preserved in the other taxa, except in *Tepexisaurus*, in which they are not well defined). *Electroscincus* may be distinguished from *Retinosaurus* and *Tepexisaurus* by the presence of imbricate, cycloid scales on the dorsum, rather than tuberculate, juxtaposed scales.

*Electroscincus*, *Tepexisaurus*, and *Retinosaurus* have the same primitive lepidosaurian manual phalangeal formula, and *Electroscincus* and *Tepexisaurus* share the same primitive lepidosaurian pedal phalangeal formula (posterior limbs not preserved in *Retinosaurus*), but *Electroscincus* and *Retinosaurus* may be distinguished from *Tepexisaurus* by having narrower ungual phalanges (unguals of *Tepexisaurus* are about three times the width of the penultimate phalanges while in *Electroscincius* these phalanges are subequal in diameter).

Recent time-calibrated phylogenies report that crown-scincids began diversifying in the Mid-Cretaceous, and so chronologically, *Electroscincus* may belong within crown-group Scincidae, though the huge diversity of living skinks, and current lack of consistent diagnostic features for the major groups thereof, as well as the limited material present in this fossil currently precludes any further diagnosis of *Electroscincus* within Pan-Scincidae. Nonetheless, *Electroscincus* can be differentiated from many extant groups that are limbless, or limb attenuated, including all Acontinae, and many genera of the other recognized subfamilies, particularly Scincinae and Sphenomorphinae, which have a high proportion of limb-reduced or attenuate taxa.

**Figure 4:**
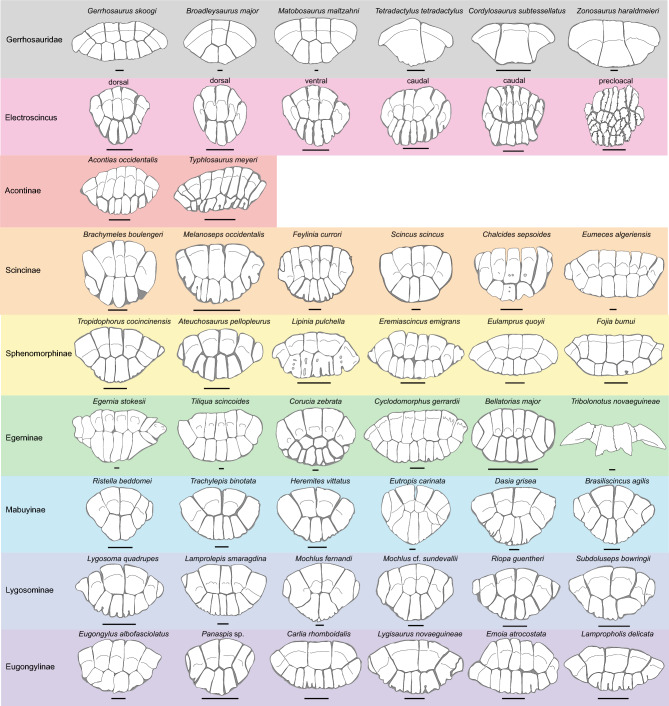
Schematic representations of scincoid osteoderms, depicting the osteodermite arrangement in *Electroscincus zedi,* the gular osteoderms of gerrhosaurs and the nuchal osteoderms of representative genera from all scincid subfamilies. Scale bar equals 0.5mm.

Considering the body osteoderms (Fig. 4), the number of osteodermites (anterior, middle and posterior) in *Electroscincus* is similar to many skinks in our sample, including *Melanoseps occidentalis* and *Scincus scincus* (Scincinae), *Lipinia pulchella* and *Eulamprus quoyii* (Sphenomorphinae), *Ristella beddomei* (Mabuyinae), and *Eugongylus albofasciolatus* and *Lampropholis delicata* (Eugongylinae), and differs considerably from any individuals of Gerrhosauridae, Acontinae, Egerninae and Lygosominae that we examined.

### Phylogenetic placement

**Figure 5:**
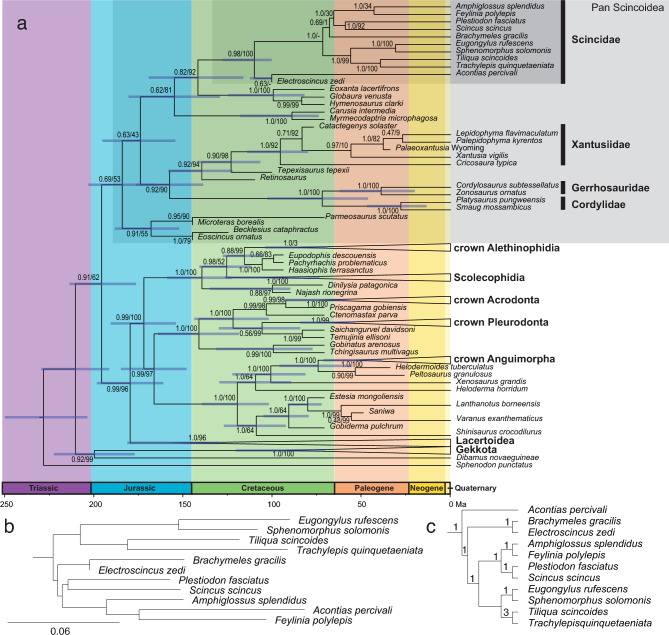
(**a**) Bayesian timetree of Squamata based on a combined molecular/morphological data set. Posterior probability and bootstrap support values are given at key nodes. Phylogenetic position of *Electroscincus zedi* in the Maximun Likelihood (**b**) and parsimony (**c**) analyses. Bremer support values on the nodes of the parsimony analysis.

Our phylogenetic analyses (Fig. 5) recover *Electroscincus zedi* in different positions depending on the optimality criterion used, but in all analyses it is recovered as member of the Pan-Scincoidea. While many fossil Pan-scincoid taxa are deep stem lineages, *Electroscincus zedi* is instead recovered consistently grouped within scincids: as sister to acontine skins in the Bayesian timetree analysis (Fig. 5a) with moderate nodal support, as sister to *Brachymeles* in the Maximum Likelihood analysis with high support (Fig. 5b), or nested among non-acontine skinks in the parsimony analysis, although with Bremer support of 1 (Fig. 5c).

Parsimony results are less well resolved, but included five synapomorphies shared with other members of Scincidae: (1) compound osteoderms in dorsal scales (known also in two gerrhosaurids), (2) compound osteoderms in ventral scales (shared also with gerrhosaurids and the fossil *Parmeosaurus scutatus*), (3) dorsal body scales cycloid (rounded and overlapping), (4) lateral body scales imbricate, overlapping, and (5) ventral scales same size as adjacent laterals. In the Bayesian results, there are nine morphological synapomorphies for the crown group Scincidae, unfortunately none of them are observable in the *Electroscincus*. In the Maximum Likelihood analyses, there are also no morphological synapomorphies uniting *Electroscincus* and the non-acontine skinks. All the synapomorphies *Electroscincus* shares with the crown group Scincidae are from the integument and cannot, therefore, be evaluated in many fossil taxa in which  skeletal features but not osteoderms are preserved.

The timetree analysis estimates the divergence between *Electroscincus* and extant scincids to have occurred in the Cretaceous, approximately 125 Ma. Scincidae (including *Electroscincus*) is recovered as most closely related to a clade formed by the extinct taxa *Hymenosaurus*, *Globaura*, and *Eoxanta*, but with only moderate nodal support. In addition to Pan-Scincoidea, other major squamate clades recovered as monophyletic include Anguimorpha, Gekkota, Iguania, Lacertoidea, and Serpentes. Scinciformata *sensu* Brownstein et al.^21^ is not monophyletic, as we recover Lacertoidea to be more closely related to Toxicofera (Anguimorpha + Iguania + Serpentes) than to Pan-Scincoidea. Our combined analysis of molecules and morphology recovers a pattern of relationships that matches previous studies based on molecules^15,18,19^.

### Holotype

Peretti Museum Foundation/ GRS GemResearch Swisslab AG (GRS-Ref-51036).

Type locality. Specimen comes from mid-Cretaceous (Late Albian/early Cenomanian) outcrops in the Myitkyina District, Hukawng Valley, Kachin Province, northern Myanmar, approximately 100 km west of the town of Myitkyina. Precise location of these mines, history of excavations, and stratigraphy of the Burmese amber deposits are summarized elsewhere^51^.

Etymology. The generic name is a combination of the Latin word for amber (*electrum*) and skink (*scincus*). The species epithet *zedi* refers to the bell-shaped stupas that house relics at Burmese Buddhist temples, referencing the smooth-sided amber housing the fossil remains, while also honoring David Temple, Curator of Paleontology at the Houston Museum of Natural Sciences (HMNS), for his contributions to palaeontology and public awareness of Burmite fossils (including the social conflict associated with its mining in Myanmar) by developing the exhibit “Amber Secrets, Feathers from the Age of Dinosaurs”.

### Description of the skeleton

**Figure 6:**
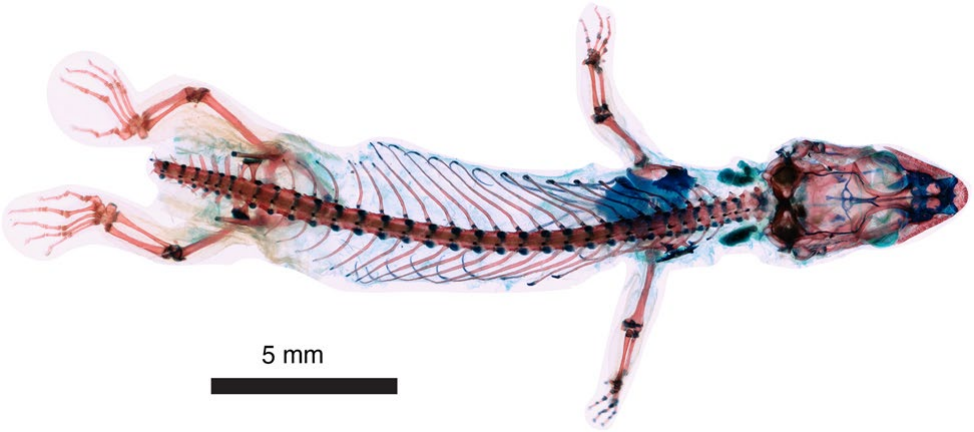
Cleared and stained subadult specimen of the sphaerodactylid gecko *Sphaerodactylus townsendi* from the University of Puerto Rico, Río Piedras Collection (UPRRP 006400). Note that the pedes exhibit proportionally less cartilage (blue) than the manūs. Photograph courtesy of Elyse Howerton.

The piece of amber includes two disconnected parts of the lizard, containing the scales and mostly appendicular bones, but is clearly part of a single individual (Figs. 2, 3). The degree of ossification of limb bones indicates that it is an adult or subadult; the leg being more completely ossified than the arm, as evidenced by the morphology of the phalanges and potentially indicating differential timing in limb formation (Fig. 3), which occurs in subadults of living species of lizards (Fig. 6). The right scapulocoracoid is large, well preserved, has a well-defined glenoid, and a short, wide scapula, with a dorsal expansion nearly identical to the width at the level of the glenoid fossa. The scapulocoracoid is surmounted by a greatly expanded cartilaginous (as indicated by its reduced radio-opacity) suprascapula, with a maximum width approximately twice that of its junction with the scapula. The coracoid has a well-defined posterior coracoid emargination. The interclavicle is cruciform, having a short anterior process extending cranial to the lateral processes. Each lateral process is as long as the posterior process.

The autopod-zeugopod-stylopod ratios are 6:5:6 (anterior limb) and 10:5:7 (posterior limb). The right humerus has a well-defined head and is expanded distally. The radius and ulna are preserved, but hard to distinguish from one another. The ulna possesses a distinct olecranon process. Manual metacarpals and phalanges exhibit incomplete ossification at the metacarpal-phalangeal, and inter-phalangeal joints. Metacarpals longer than proximal phalanges. The phalangeal formula of manus is difficult to determine, as there is not a complete set of phalanges in either hand, but the phalangeal complement seems to be 2:3:4:5:? Given that the primitive pedal formula is conserved, we expect that the manual formula will also be that primitive for squamates, 2:3:4:5:3. Penultimate phalanges in the hand are longer than the antepenultimate. The right leg is better preserved than the left, including femur, tibia and fibula, and all the tarsals and phalanges. The femur has a visible internal trochanter. It is common that many skinks, for example mabuyine lineages, exhibit short zeugopodal segments when compared with other parts of the limb, this is also the case in *E. zedi*. Metatarsals are longer than the proximal phalanges. The phalangeal formula of the pes is 2:3:4:5:4, with pedal digits straight, ungual phalanges narrow and claws not strongly recurved.

The tail segment includes four vertebrae, the first three have long transverse processes (about three times longer than the centrum width) and are autotomic. The first vertebra preserves only the posterior segment, the second and third are complete and show some faint indication of the fracture plane, and the fourth has a well defined fracture plane. The transverse processes are formed by two lateral laminae that join distally, forming a gap near the centrum, the fracture planes pass through the transverse process as has been reported for the skink *Plestiodon fasciatus*^52^. The size of the transverse process is reduced abruptly between the third (three times the centrun width) and fourth (same length as the centrum width) vertebrae. The centrum is notochordal and there is one complete “V”-shaped chevron articulated between the third and fourth preserved vertebrae.

### Description of osteoderms

In *Electroscincus,* the osteoderms take the form of the strongly imbricating cycloid scales of the body in which they are embedded, and the overlap of adjacent contacting scales, results in a corresponding overlap of the osteoderms that is clearly revealed by the CT scans. Osteoderms covering the body are ovoid (wider than long) and patterned in a staggered arrangement, so that the longest part of each osteoderm overlies the two osteoderms posterior to it. The osteoderms are compound, being formed by several smaller bony plates or osteodermites. The arrangement of osteodermites varies across the body of the fossil; the trunk osteoderms have five anterior and three to five posterior osteodermites, the caudal osteoderms have six to eight anterior and six posterior osteodermites, the precloacal osteoderms have nine anterior, 11 posterior and 13 interior osteodermites. Since each osteoderm has multiple rows of osteodermites, the posterior osteodermites of the anterior osteoderm, overlap the anterior osteodermites of the posterior osteoderm. The anterior osteodermites are slightly thickened anteriorly, forming a flattened ridge that articulates with the posterior osteodermites of the preceding osteroderm. Osteoderms in the limbs (Fig. 3) are smaller (mean length 0.253 mm, n = 5) than the body osteoderms (mean length 1.028 mm, n = 8), averaging 24.6% the length of the body osteoderms.

Scincid compound osteoderms vary significantly in both the number of osteodermite rows, and the number of osteodermites per row (Fig. 4). Osteoderms from the nuchal region are usually comprised of an anterior and a posterior row of osteodermites. Of all the species observed in this study, only egernine skinks such as *Tribolonotus novaeguineae, Corucia zebrata* and  *Cyclodomorphus celatus* deviate from this pattern. Caudal osteoderms often comprise three rows of osteodermites with the anterior row having a lower number than the posterior rows^53^.

Regarding flexibility, it has been demonstrated that squamates with fused osteoderms have a stiffer skin than squamates with compound osteoderms^54^. In *Electroscincus* the area anterior to the cloaca is covered by osteoderms formed by more numerous and smaller osteodermites than in the rest of the body, therefore it is very likely that in life the skin covering this area was more pliable than in other regions.

## Discussion

The Cretaceous period is an important time for the diversification for squamates^20,55,56^; squamates, other tetrapods, arthropods, and angiosperms may have all been affected by a large scale macroevolutionary process known as the Cretaceous Terrestrial Revolution, although it has been determined that at least for squamates, phylogenetic diversification and the ecological roles of the main constituent clades were established in the Jurassic^57^. During the mid-Cretaceous, there was probably an expansion of ecological guilds among squamates, as indicated by an increase in the diversity of fossil groups^58^, dentitional disparity^59^, and cranial diversity^60^. The mines of northern Myanmar, the world's largest amber deposit of squamates, preserve  this diversity  ^61^, documenting a critical period of diversification of many of the major extant squamate clades^58^, consistent with molecular clock estimates^18,62^. Alternatively, the increase in diversity of fossil groups during this period could be due to a sampling bias, especially in localities that facilitate preservation of delicate structures such as the amber mines of Myanmar, or fossils from the Yixian Formation of Liaoning, China^68^.

To be useful, the phylogenetic position of fossils used to calibrate modern time trees must be linked to modern groups, either directly by phylogenetic analyses or apomorphy based diagnoses^63^. With adequate calibration, time trees provide estimates of historical diversification processes in groups that may fossilize poorly. In the case of skinks, previous crown age estimates for the family range from 75 to 118 MYA, averaging 94 MYA^64^. Although our phylogenetic analyses were inconsistent with respect to the position of *E. zedi* within the crown group Scincidae, its Cenomanian age is squarely at the center of the estimated range for the diversification of crown Scincidae^2,65^.

**Figure 7:**
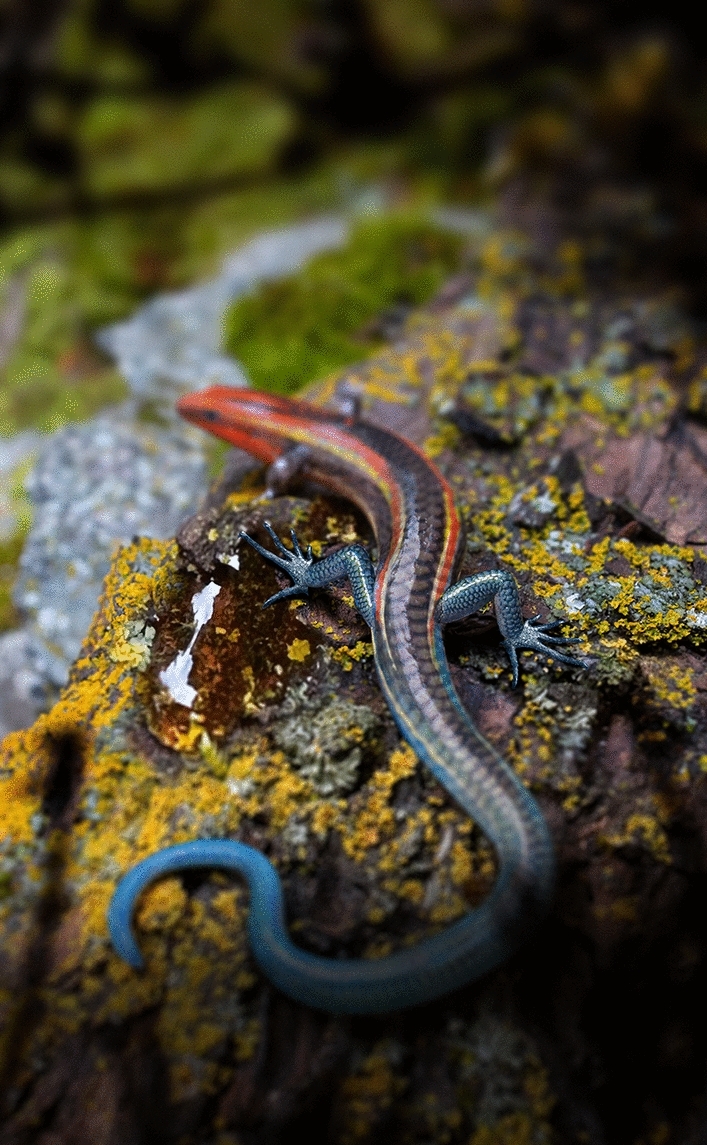
Life reconstruction of *Electroscincus zedi*. Areas of the lizard not represented in the material available are depicted as blurred. Illustration by Stephanie Abramowicz.

*Electroscincus* is the only known squamate fossil that possesses unambiguously cycloid osteoderms, and its external appearance, especially the feet and body, resembles modern skinks (Fig. 7). Cycloid osteoderms were reported in the Early Cretaceous *Scandensia cervensis*^66^, in which they vary from large and somewhat angular dorsally to smaller and more ovoid ventrally. Although similar in shape to the cycloid osteoderms of skinks, including *E. zedi*, they are not compound. Some other fossils in amber from Myanmar have been considered potential scincomorphs^58^, including one specimen (JZC Bu269) having cycloid scales in the postocular region. However, the scales of this specimen are multicarinate, juxtaposed and surrounded by tiny granular scales rather than imbricate ones, and CT scans of this specimen did not reveal any mineralized material associated with, or underlying, these scales.

Among extant squamates, the presence of compound osteoderms on the dorsum is diagnostic for the family Scincidae and two species of gerrhosaurids. Compound osteoderms are exceedingly rare in the fossil record, since these osteoderms comprise small, delicate osteodermites, which disarticulate easily and may become lost during the fossilization processes, or may be inadvertently removed in specimen preparation^66^. Compound osteoderms require special taphonomic conditions to be preserved, in an analogous way to other equally diagnostic integumentary structures of archosaurs, such as feathers^67^. Amber is one of the best natural preservation media for integument, preserving even fine structures such as compound osteoderms or tiny scales in miniaturized geckos^58,68^. Amber offers similar conditions (quick burial and anoxia/hypoxia) to lagerstätten and aeolian deposits of the Campanian Gobi Desert that have preserved remarkably complete skeletons with associated fine integumentary structures^69–71^.

Osteoderms have evolved independently at least eight times in squamates^72^, and some of the oldest squamates with osteoderms covering most of their bodies include paramacellodids, which are known from the Middle Jurassic. Although their phylogenetic placement is uncertain, it has been suggested that they represent stem Scincoidea^20,50^. It is likely that osteoderms evolved once in basal scincoideans, and that their absence in Xantusiidae^22^ represents a rare instance of secondary loss of osteoderms in the squamate tree of life (complete loss of osteoderms have also been reported in some species of varanids^73^). The order and timing of the evolution of compound and simple osteoderms in this group is less clear; simple osteoderms are reported from all major modern and fossil scincoid lineages, excluding Xantusiidae, and are likely to be plesiomorphic for Pan-Scincoidea. Ventral compound osteoderms either evolved twice—in Scincidae and Gerrhosauridae—or, equally parsimoniously, compound osteoderms may be plesiomorphic for Scincoidea, completely lost in Xantusiidae and only retained in the ventral regions of gerrhosaurids. Dorsal compound osteoderms are widely present in Scincidae, but only known from two species of gerrhosaurids (and the arrangement of osteodermites in these taxa is quite different from that seen in the compound osteoderms of other modern scincoids Fig. 2^74,75^).

Molecular clock estimates predict the presence of Scincidae during the Cretaceous^18,50^ and the presence of a lizard with compound osteoderms confirms this estimate and reinforces the idea of early diversification of armor among squamates. Osteoderms are generally assumed to have evolved principally for protection, but other functions like thermoregulation, lactate sequestration, and calcium storage, have been suggested (^50^ and references therein). Biomechanical experiments also indicate that the different configurations of osteoderms yield differential loading properties, and the presence of compound osteoderms likely reduces overall stiffness^54^. The reduction of the size and number of simple osteoderms in the inguinal, axillary and cloacal regions of heavily armored cordylids (e.g. *Cordylus*, *Namazonurus,* and *Smaug*)^49^ and anguimorphs (*Celestus*, *Elgaria*, *Pseudopus,* and the fossil *Ophisauriscus*)^76,77^ suggests that these structures may impede the free movement of limbs and the cloacal region. The same regions in skinks retain their imbricate compound osteoderms, without any apparent reduction in flexibility. The evolution of extensive overlapping compound osteoderms in early skinks like *Electroscincus* produced a flexible dermal armor and may have been a key innovation that facilitated the remarkable diversification of the family. 

Although diagnoses should ideally employ autapomorphic characters, the material preserved imposes a limitation in this regard. However, *Electroscincus* is allocated to Scincidae on the basis of a combination of integumentary characters and is easily differentiated from other Cretaceous taxa previously referred to Scincoidea by its morphology and geographical distribution. It is certainly more complete than many fossil taxa described on the basis of fewer bones, or footprint impressions. Given that it retains at least some of the postcranial skeleton and skin impressions, in addition to the osteoderms, it is also more complete than many amber inclusions, which are often represented by skin impressions only^58^. It is uncertain if more specimens of skinks in amber will become available in the near future (See ethics statement), but if so, *Electroscincus* possesses a number of features that could be meaningfully compared with them. It also serves as a critical fossil to document the early origin of dermal armor in this megadiverse group of squamates.

## Methods

### Ethics statement and specimen chain of custody

The specimen described in this study was purchased by Dr. Ru Smith in March of 2016, more than one year prior to the June 2017 military takeover of the Myanmar mining regions, which has been established as the cutoff date for conflict amber in the guidelines of the Society of Vertebrate Paleontology (SVP) Myanmar Working Group. Dr. Smith gifted this fossil to Juan D. Daza in 2017, and the specimen was donated to the Peretti Museum Foundation, where it is now cataloged and available for examination.

We followed the SVP recommendations for researchers, research institutions and publishers available at https://vertpaleo.org, particularly the recommendations for material acquired before 2017. Detailed confidential information about the history of the specimen is available upon request, given the situation of Myanmar, the information about the vendor and previous owners is confidential, but is associated to the specimen in the catalogue of the Peretti Museum Foundation (PMF.org). We fulfill four of the recommendations from SVP working group: (1) Material was acquired from a seller that is not on the list of banned persons by the United Nations Human Rights Council (42 session, 2019, A/HRC/42/L.21/Rev.1), (2) The material is housed at the Peretti Museum Foundation which is a repository registered under the Swiss Government, (3) The material is available to researchers at the PMF and freely accessible as a digital copy at www.morphosource.org, (4) The PMF catalogue includes proof of current and previous ownership with specific dates and is supported by photographic metadata.

### Digital photographs and X-rays

Both sides of the specimen were photographed using the 3D stitching function on a Keyence Digital Microscope VHX-7000 series. In all photos the glare was removed digitally, and the specimen was illuminated using specular reflection and transmitted light. Digital X-rays of the specimen were obtained using Thermo Scientific™ PXS5-927 microfocus X-ray source and the Mars 1717 V Wireless a-Si Flat Panel Detector. A radiopaque ruler was included on the X-rays for the scale.

### Microtomography

The specimen was CT scanned at the University of Florida’s Nanoscale Research Facility, using a ZEISS Versa 620 X-ray Microscope. Two initial scout-scans using a panel detector and geometric magnification imaged the fore and hind body at a voxel resolution of 9.619 µm, which allowed a second series of scans using a 4X objective to image the cloaca and hindlimb at a resolution of 2.296 µm. The X-ray tube voltage and current were set at 80 kV and 125 µA respectively and the detector capture time were modified to maintain sufficient X-ray intensity to produce clear images (0.16 s for the flat panel and 16 s for the objective). Radiographs were converted into tomograms using the XMReconstructor (Carl Zeiss) software suite, and renderings of the osteoderms and appendicular bones were generated using VGStudioMax 2023.2 (Volume Graphics, Heidelberg, Germany).

### Phylogeny

A combined morphological-molecular data set was constructed for the phylogenetic analysis. Taxon sampling includes 113 representative squamates plus *Sphenodon* (Data S1-S2). Both molecular and morphological data are included for the 83 extant taxa included in the data set; a further 31 extinct squamates (including the focal taxon) have morphological data only. Six hundred ninety-one morphological characters were obtained from published data sets^17,20,21^. The focal taxon was scorable for 59 of these characters. DNA sequence data (52 genes, 42,311 positions) were obtained from a published molecular matrix^18^. Morphological data were partitioned into ordered and unordered subsets according to the scoring employed by Gauthier et al.^20^ and Reeder et al.^17^. ModelFinder in IQ-TREE 2.2^78,79^ (Available at: http://www.iqtree.org) was used to identify the best-fitting molecular partitioning scheme and models under the Bayesian Information Criterion. A maximum likelihood phylogenetic analysis was performed in IQ-TREE 2.2, using the partitions and model parameters identified in the preceding step, and assessing branch support with 1000 ultrafast bootstrap replicates. A Bayesian timetree analysis was performed in BEAST 2.7.3 ref^80^ (Available at: https://www.beast2.org) using the fossilized birth-death tree model and dated tips. Partition and model parameters for the morphological and molecular characters were set as for the likelihood analysis. The analysis was performed as two independent runs of 200 million generations, sampled every 10000 generations, with the first 10 percent of samples discarded as burn-in. Run parameters were visualized in Tracer 1.7.3 ref^81^ to ensure adequate chain length. Post-burnin samples from the independent runs were combined using LogCombiner 2.7, and a maximum clade credibility tree generated using TreeAnnotator 2.7.4.

The data matrix was analyzed using parsimony as the optimality criterion with the software TNT version 1.6 ref^82^ (Available at: https://www.lillo.org.ar/phylogeny/tnt/), using *Sphenodon punctatus* as outgroup. The search was performed until 20 hits of minimum length were found. Each hit was run using 20 random addition sequences, each one subject to sectorial searches, ratchet, and tree drift, and fusing trees every 5 sequences^83,84^. Bremer support values^85^ were calculated using TBR swapping, keeping trees up to 10 steps longer.

### Zoobank registration

This publication and its nomenclatural have been registered in ZooBank, the proposed online registration system for the International Code of Zoological Nomenclature (ICZN). The ZooBank LSIDs (Life Science Identifiers) for this publication are: urn:lsid:zoobank.org:pub: 2B3C8010-4046-422B-953B-34B44258F80D (publication), urn:lsid:zoobank.org:act: 9657A3A9-A801-43AA-9F80-6B88D9A69135 (†*Electroscincus zedi*).

### Supplementary Information

Supplementary Information 1.

Supplementary Information 2.

Supplementary Information 3.

Supplementary Information 4.

Supplementary Information 5.

## Data Availability

All data are available in the main paper or the supplementary materials. The holotype of *Electroscincus zedi* (GRS-Ref-51036) is housed at the Peretti Museum Foundation, Switzerland. The tomographic data set of the fossil is available at MorphoSource (www.morphosource.org), specimen ID: Specimen 000606852. Specimen renderings and scans are available at the following links: Anterior body 10.17602/M2/M606860 Posterior body 10.17602/M2/M606861 Hind limb osteoderms 10.17602/M2/M606866 Cloacal osteoderms 10.17602/M2/M606870.
